# From global to national GHG budgets: the REgional Carbon Cycle Assessment and Processes-3 (RECCAP3)

**DOI:** 10.1093/nsr/nwaf037

**Published:** 2025-02-07

**Authors:** Josep G Canadell, Benjamin Poulter, Ana Bastos, Philippe Ciais, Judith Hauck, Robbie Andrew, Pierre Friedlingstein, Giacomo Grassi, Robert B Jackson, Jens Daniel Müller, Corinne Le Quéré, Michael O'Sullivan, Prabir Patra, Glen P Peters, Julia Pongratz, Marielle Saunois, Stephen Sitch, Hanqin Tian, Yohanna Villalobos, Xuhui Wang

**Affiliations:** Global Carbon Project, CSIRO Environment, Australia; Biospheric Sciences Laboratory, NASA Goddard Space Flight Center, USA; Institute for Earth System Science and Remote Sensing, Leipzig University, Germany; Laboratoire des Sciences du Climat et de l'Environnement, LSCE/IPSL, CEA-CNRS-UVSQ, Université Paris-Saclay, France; Alfred-Wegener-Institut, Helmholtz-Zentrum für Polar- und Meeresforschung, Bremerhaven Germany and Universität Bremen, Germany; CICERO Center for International Climate Research, Norway; Faculty of Environment, Science and Economy, University of Exeter, UK; Laboratoire de Météorologie Dynamique, Institut Pierre-Simon Laplace, CNRS, Ecole Normale Supérieure, Sorbonne Université, Ecole Polytechnique, France; European Commission, Joint Research Centre (JRC), Italy; Department of Earth System Science, Woods Institute for the Environment, and Precourt Institute for Energy, Stanford University, USA; Environmental Physics, Institute of Biogeochemistry and Pollutant Dynamics, ETH Zurich, Switzerland; Tyndall Centre for Climate Change Research, School of Environmental Sciences, University of East Anglia, UK; Faculty of Environment, Science and Economy, University of Exeter, UK; Research Institute for Global Change, JAMSTEC, and Research Institute for Humanity and Nature, Japan; CICERO Center for International Climate Research, Norway; Ludwig-Maximilians-Universität München, Germany; Max Planck Institute for Meteorology, Germany; Laboratoire des Sciences du Climat et de l'Environnement, LSCE/IPSL, CEA-CNRS-UVSQ, Université Paris-Saclay, France; Faculty of Environment, Science and Economy, University of Exeter, UK; Center for Earth System Science and Global Sustainability, Schiller Institute for Integrated Science and Society, Boston College, USA, and Department of Earth and Environmental Sciences, Boston College, USA; Global Carbon Project, CSIRO Environment, Australia; Lund University, Sweden; College of Urban and Environmental Sciences, Institute of Carbon Neutrality, Sino-French Institute for Earth System Sciences, College of Urban and Environmental Sciences, Peking University, China

As human-induced climate change intensifies, there is a growing demand for more comprehensive greenhouse gas (GHG) intelligence to support the development and implementation of mitigation policies. This demand is amplified by the surge in GHG data availability, best illustrated by the proliferation of GHG monitoring satellites being launched, and by the increase in GHG reporting requirements for all countries under the Enhanced Transparency Framework of the Paris Agreement.

Globally, comprehensive GHG budgets with the skill and detail to separate natural and anthropogenic GHG sources and sinks have played a critical role in supporting the development of global decarbonization pathways. Now, this type of comprehensive earth system budget is required at the national scale, where climate and energy policies are implemented, with an increasing reliance on natural carbon sinks to achieve net zero emission targets. This approach will link the global requirements for climate stabilization with national-scale mitigation efforts and biospheric changes.

The Global Carbon Project (GCP) is developing and seeking involvement in the Regional Carbon Cycle Assessment and Processes-3 (RECCAP3) to support the development of comprehensive national-scale GHG budgets, including natural and anthropogenic sources and sinks of the three most important GHGs: carbon dioxide (CO_2_), methane (CH_4_) and nitrous oxide (N_2_O).

This initiative builds on two decades of progress in developing global budgets for CO_2_, CH_4_ and N_2_O [1–3]. It also follows two prior RECCAP efforts: RECCAP1 [4] and RECCAP2 [5–7]. These earlier efforts produced regional GHG budgets covering 10 land regions and 5 ocean regions, providing a more detailed and accurate picture of regional GHG fluxes than ever before.

RECCAP3 will focus on national budgets and integrate multiple flux estimates using independent approaches involving observational data sets and national GHG inventories, biogeochemical modeling, and advanced analytical and AI tools to harness the rapidly expanding availability of ground-based, satellite and activity data. This information will be tailored to the national circumstances of each country. RECCAP3 will also continue to leverage atmospheric-based GHG flux estimates from atmospheric inversion modeling to better constrain surface fluxes. We acknowledge the limitations to using atmospheric inverse modeling for small countries given the current sparse network of near-surface observations and the coarse resolution of satellite-based column CO_2_ and CH_4_. However, attempts are being made to increase the density of surface measurements, including the deployment of inexpensive sensors. National programs are also being established to increase the number of observations that make it possible for atmospheric inversions to be useful in geographically complex and relatively small countries such as New Zealand [8]. RECCAP3 will not be able to cover all countries and will have an initial focus on larger countries and those with the potential for deploying additional measurements. Global systems capable of producing less comprehensive national budgets with additional benchmarking are also in scope, which, with their limitations, will be able to cover more countries at regular intervals [9].

**Figure 1.**
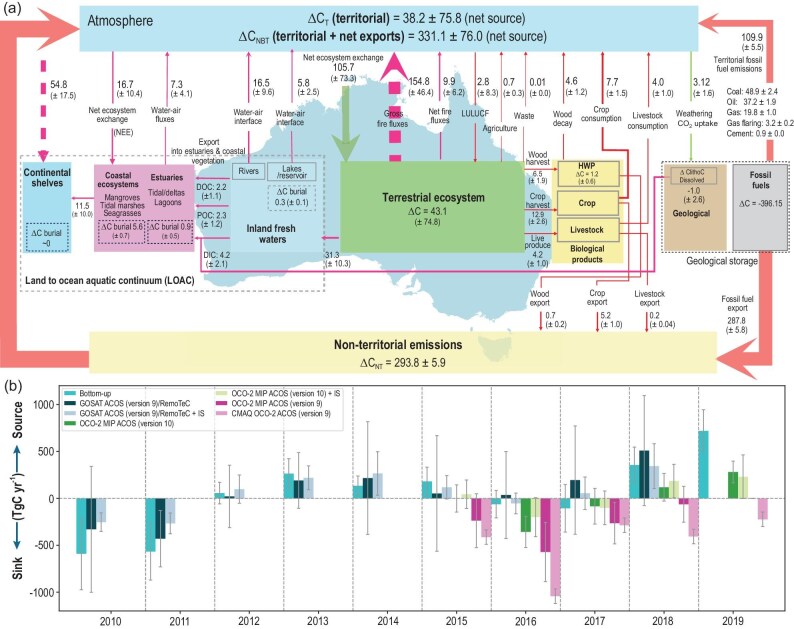
An illustrative example of the type and detail for country GHG budgets that RECCAP3 intends to develop for many countries. (a) Annual mean decadal (MtC y^-1^) bottom-up Australian carbon budget for 2010–2019; green arrows represent natural fluxes, red arrows are anthropogenic fluxes and magenta arrows are a mix of natural and anthropogenic fluxes. (b) Annual top-down (atmospheric inversions) Australian carbon balance for 2010–2019; different color bars represent different inversion frameworks and versions of atmospheric retrievals by the Greenhouse gas observing satellite (GOSAT) and the Orbiting carbon observatory-2 (OCO-2), and dark blue represents the bottom-up estimates (adapted from [13]).

The reconciliation of independent and semi-independent bottom-up and top-down flux estimates will enhance the robustness and confidence of the GHG estimates. Figure 1 and Yuan *et al.* 2025 [10] are examples of pioneering top-down and bottom-up national comprehensive GHG budgets, for Australia and China, respectively.

Here, we outline the emerging gaps, necessary actions that a national comprehensive GHG budgeting effort aims to address, and critical operational and capacity-building aspects to ensure its success as a globally coordinated effort.

**The exponential growth of GHG data and monitoring systems.** The rapid growth in GHG data provision and monitoring systems developed and run by agencies and programs outside of national users poses an increasing challenge. There is no more striking example of the rapid growth in GHG information than the efforts by space agencies and the private sector to deliver data on GHG concentrations, land cover and aboveground biomass derived from satellites at unprecedented spatial and temporal resolution. In addition, the Global Greenhouse Gas Watch initiative of the World Meteorological Organization (WMO 2024) and other non-governmental organizations are coordinating the deployment of multiple global and regional monitoring systems that will flood nations with quasi near-real-time GHG information. A national capacity needs to be developed to interpret and assess the quality of these global products for country-level applications and to attribute changes in GHG fluxes to natural and anthropogenic drivers. RECCAP3 aims to enhance the national capability to evaluate new data for national applications, assess their uncertainties and integrate with existing domestic data and monitoring systems. We acknowledge the decline of some terrestrial, ocean and atmospheric observational networks, such as the surface ocean carbon observations over the past two decades, underscoring the urgent need to reverse this trend.

**The gap between national GHG inventories and global GHG budgets.** A significant discrepancy exists between definitions for land-based emissions used in national greenhouse gas inventories (NGHGIs), reported under the Paris Agreement, and those employed to develop global carbon budgets and decarbonization pathways by the GCP and the Intergovernmental Panel on Climate Change (IPCC).

This discrepancy emerges from the different definitions of what constitutes natural versus anthropogenic carbon sinks. Global carbon budgets and decarbonization pathways consider an anthropogenic sink to be the CO_2_ removal driven by the direct actions of humans, such as a reforestation program, but excludes the indirect effects due to increased atmospheric CO_2_ (i.e. CO_2_ fertilization effect on plant growth) and climate changes (e.g. lengthening of the growing season in high latitude ecosystems). NGHGI defines an anthropogenic CO_2_ sink as all removals occurring in pre-determined managed lands. That includes the CO_2_ removal from direct human actions, such as reforestation, and the indirect effects of CO_2_ and climate changes on the growth of those new forests. These varying definitions stem primarily from the distinct initial objectives of each approach and community, as well as the specialized tools and accounting frameworks developed to meet those objectives.

This discrepancy has led to a difference of 6.7 GtCO₂ per year between the two approaches over the past two decades [11], with NGHGIs showing an aggregated global sink of 1.9 GtCO_2_yr^−1^ while global modeling shows a net source to the atmosphere of 4.8 GtCO_2_yr^−1^. Without harmonizing these two different approaches, national mitigation targets calculated using NGHGI methods risk leading to higher warming trajectories than the IPCC-assessed decarbonization scenarios [12]. A national focus will enable the development of a comprehensive carbon accounting, which captures both direct sources and sinks (e.g. deforestation, fossil fuel combustion) and indirect human-driven impacts (e.g. CO_2_ fertilization effects on photosynthesis, warming-induced increases in wetland CH_4_ emissions, and increased riverine GHG emissions from land clearing and warming temperatures). This comprehensive accounting can then be compared to the NGHGI to estimate the magnitude of the gap at the national level and adjust accordingly the national contributions and the global decarbonization pathways.

**Expanding the coverage of GHG sources and sinks.** There are GHG sources and sinks that currently are not accounted for in both NGHGIs and scientific GHG budgets due to limited observations and the lack of suitable modeling tools. RECCAP3 will facilitate high-resolution model development and the uptake of new data, including fluxes from inland freshwaters (e.g. lakes, reservoirs, rivers, farm ponds and wetlands) and their partition between anthropogenic and natural origins. It will also include the CO_2_ sink from cement carbonation and fluxes from reported carbon-dioxide-removal (CDR) activities. For countries with coastlines, national efforts will include fluxes from coastal ecosystems like estuaries, mangroves, salt marshes and seagrass meadows. The growing interest in human-driven carbon sequestration potential in coastal oceans requires the accurate estimation of baseline fluxes from which enhanced human-driven sequestration can be measured. Thus, RECCAP3 will further develop water–air GHG flux estimates for continental shelves and exclusive economic zones (EEZs), which typically extend up to 200 nautical miles from a country's coastline.

**Oceans and regions of special interest.** RECCAP3 will maintain its emphasis on global and regional ocean assessments while addressing emerging issues, such as the changing biological carbon pump and the land–ocean aquatic continuum (e.g. enhanced carbon input from rivers and coastal erosion) that connects the land and ocean carbon cycles and is experiencing rapid changes. In addition, RECCAP3 will identify key regions on land and in the ocean undergoing rapid changes in GHG sources and sinks that can lead to significant biogeochemical-climate feedbacks and require a broader regional approach. Notable regions include the circumpolar permafrost region, the Coastal Ocean, tropical forests (Amazonia, Congo Basin, Indonesia archipelago) and the role of semi-arid regions in the variability, trend and size of global CO_2_ sources and sinks.

**Operational aspects and capacity building.** RECCAP3 is designed to be developed in multiple phases over the coming decade. The GCP will provide coordination while fostering the development of a community of practice to lead the development of country-specific GHG budgets. Syntheses of budgets will contribute to the Global Stocktake of the Paris Agreement. RECCAP3 will emphasize capacity building and nurturing a new generation of scientists from both developed and developing countries. It will also integrate and compare data from the NGHGIs, which requires developing the necessary expertise and collaboration between NGHGI compilers and the broader scientific community.

Tracking comprehensive country-level GHG budgets will support the alignment of national reporting with global decarbonization requirements and identify actionable insights to improve national reporting. RECCAP3 will also monitor changes in natural terrestrial and ocean GHG sources and sinks, enabling the early detection of shifts in current and emerging biogeochemical-climate feedbacks.
